# Reception Centers and Practices for Women Migrants

**DOI:** 10.12688/openreseurope.18207.1

**Published:** 2024-09-09

**Authors:** Marie Ruiz, Stellamarina Donato

**Affiliations:** 1 Universite de Picardie Jules Verne, Amiens, Hauts-de-France, France; 2 Libera Universita Maria Santissima Assunta, Rome, Lazio, Italy

**Keywords:** Migration, gender, reception, policy

## Abstract

This paper focuses on reception centers for women migrants from a gender mainstreaming perspective and it reflects on the current practices and their adequacy with women migrants’ needs. Based on COST Action Women on the Move (CA19112)’s experience and research, it discusses the different typologies of reception facilities, and highlights the impact of reception on women migrants’ integration, as well as the necessity to coordinate best practices across Europe and EU Member States. Then authors recommend to improve the quality of reception with better trained staff and measures adequate to women migrants’ specific needs for their safety and health. The research also suggests introducing integrative programs and coordinating information and good practices across Europe, but above all an end to detention centers for migrants.

## Disclaimer

The views expressed in this article are those of the author(s). Publication in Open Research Europe does not imply endorsement of the European Commission.

## Introduction

According to the UNHCR, before the war in Ukraine 49% of forcibly displaced people worldwide were women and girls ( UNHCR, 2022), yet the proportion has dramatically increased since February 2022, women and girls from Ukraine now accounting for 60% of refugees in the EU ( Council of Europe Report, 2022). Hence, WEMov believes that gender mainstreaming must be systematically introduced to improve women migrants’ reception ( Bekyol *et al.*, 2016). The Council of Europe defines gender mainstreaming as an approach introduced at the 1985 UN Nairobi World Conference: “Gender mainstreaming is the (re)organisation, improvement, development and evaluation of policy processes, so that a gender equality perspective is incorporated in all policies, at all levels and at all stages, by the actors normally involved in policymaking”.

This policy brief is a reflection on today’s model of reception centers and their adequacy to women migrants. It maps typologies of reception facilities for migrants, addresses the controversial parallel with concentration camps in the most inefficient cases, and proposes recommendations based on the work and research of COST Action Women on the Move (CA19112). Indeed, reception centers for migrants are regularly accused of being run like prisons ( Nethery, 2009), yet WEMov has visited facilities that respect the dignity of women migrants, and believes that these models are to be replicated and implemented more widely in the EU. The objective of this policy brief is to integrate gender mainstreaming in reception policies. Its methods are based on interviews and observations in three reception centers (the Sarajevo Temporary Residence Center (TRC) Usivak in Bosnia & Herzegovina, the Ödos Program in Spain, as well as Kutchinate in Israel, which is a day center focusing on women migrants’ integration) as well as research on women’s reception and organisations, intermediaries of women’s migration in the past and the present. This brief provides for a study on the types of assistance offered in reception centers, maps the different typologies of reception centers and raises the question of the impact of reception facilities on women migrants’ empowerment and integration, thus promoting best practices to be implemented across the EU.

## General framework

The legislative framework on gender mainstreaming and migrants’ reception at UN, EU and national levels informs us on the provisions for women migrants.

### International framework

The first reference WEMov grounds its arguments in is the Universal Declaration of Human Rights that states that “everyone has the right to a standard of living adequate for the health and well-being of himself and of his family” (UN Declaration, Article 25). This should apply to incoming migrants as the declaration guarantees the right to asylum, education, social security and suitable standards of living. The UN 1951 Geneva Convention and its 1967 New York Protocol recognize asylum as a right for applicants and an obligation for Member States (UNHCR). Against prison-like detention centers, Article 5 of the European Convention on Human Rights, the UNHCR as well as the European Court for Human Rights call for vulnerable migrants not to be treated like criminals (see Article 31 of the 1951 Convention on the non-penalisation of refugees and asylum-seekers in irregular migration).

The international framework on women’s migration was impacted by the 1995 UN World Women’s Conference in Beijing (1995), a turning point in women’s empowerment and gender equality policies. The 1979 CEDAW (Convention on the Elimination of All Forms of Discrimination against Women New York, 18 December 1979) report marked a major step towards gender mainstreaming practices introducing a ban on discriminations against women. The CEDAW Committee’s recommendations apply to undocumented women and suggests adequate assistance to Gender Based Violence (GBV) victims and vulnerable women migrants.

More recently, the United Nations’ Sustainable Development Goals (SDGs) serve as guidelines to improve women migrants’ reception and guarantee their safety and well-being. Additionally, the Council of Europe’s 2011 Istanbul Convention protects women against GBV, to be implemented in reception centers with separate wards and sanitary facilities for women and men to reduce the risks of sexual assaults. More recently, the Council of Europe’s Action Plan on Protecting Vulnerable Persons in the Context of Migration and Asylum in Europe (2021–2025) also aims at impacting reception conditions with enhanced support and services.

### European framework

In 1999, the EU introduced a Common European Asylum System (CEAS), which was reformed in 2020 to focus on 1) procedures for asylum and refoulement; 2) solidarity among member states 3) cooperation with third countries (European Commission, Common European Asylum System). The main agencies and texts that govern migration and asylum in the EU are:

• The Common European Asylum System for a quick and efficient asylum procedure

• The Reception Conditions Directive with common standards for reception conditions (housing, health, food, clothing and education) under the Charter of fundamental rights

• The Qualification Directive clarifies the asylum-seeking procedure

• The Dublin Regulation defines asylum conditions and procedure across EU States

• The Eurodac Regulation is a fingerprints system for Member States’ authorities

• The European Union Agency for Asylum assists States in the asylum-seeking process.

According to Article 13 of Directive 2003/9/EC, EU Member States are bound to provide for adequate reception conditions and humane reception, healthcare and subsistence including food, housing and clothing. Directive 2013/33/EU sets EU standards on reception for asylum seekers guaranteeing humane and dignified reception conditions ( Cameron, 2010).

For the first time in 1997, Article 3 of the Treaty of Amsterdam introduced gender equality as an EU commitments. This was confirmed by the Charter of Fundamental Rights of the European Union which framed gender equality as one of its mainstays in 2000 and the ensuing protocols and guidelines (strategy on gender equality (2001–2005) and ‘Roadmap for Equality’ (2006–2010) adopted by the European Commission. Combined with the Directive 2003/9/EC (confirmed by the Treaty of Amsterdam and the Tampere European Council) that guarantees minimum standards of dignity in migrants’ reception, women migrants’ reception is thus doubly framed by policies on gender mainstreaming and migrants’ reception.

The New Pact on Migration and Asylum, introduced in 2020 and implemented in December 2023, is meant to improve and harmonize the reception practice with increased screening of non-EU migrants, reinforced Eurodac database to fingerprint monitor non-EU migrants and more efficient asylum and refoulement procedures. The Asylum Migration Management Regulation is expected to reduce the risks of smuggling and trafficking. The Action Plan on Integration and Inclusion (2021–2027) should give non-EU migrants access to employment, housing facilities, education and health services. On 16 January this year, the Court of Justice of the European Union (CJEU) decided to recognise women as a "social group", giving them the possibility to claim refugee status for persecution and threats to which they are subject because of their gender. Yet, one major flaw has been identified in the very latest EU decision on combating violence against women (February 2024) as it does not take into account migrant women, yet regularly described at higher risks of GBV.

## Reception centers in context and practice

### Typologies of reception centers

Reception centers are not a new phenomenon: inns for travelers indeed existed in the early modern period, yet the place of women, and especially unaccompanied by a man, has always been controversial in travelling, migrating, or reception. In the 19th century age of Great Migrations, women migrants could rely on women’s networks and emigration societies to be hosted either in hostels, reception homes, schools, farms or barracks. The main issues for women were (and still are) health-related and safety. For Campesi, the reception center system’s function seems to focus on protecting local communities rather than migrants ( Campesi, 2015).

### Three reception phases

Women’s Refugee Commission has classified reception centers around 3 typologies following the arrival process: “(1) first arrival centers (or Reception and Identification Centres (RIC); (2) asylum processing centers; and (3) specialty centers” ( Women’s Refugee Commission, 2022).

(1) First arrival centers are meant to sort migrants between the “dublinable” ones and the non-asylum seekers, who will then transfer to pre-removal detention centers. The Commission notes that overcrowding lengthens the process. Belgium and Netherland’s 3-day timeframe often leads to denying accessing to asylum seekers because of limited capacity. In Germany in 2017–2021, AnkER-Centres (Centres for admittance, decision and return) were used to sort the dublinables from the non-asylum seekers and hence limit detention periods. Yet, asylum seekers sometimes had to wait for years before obtaining a stable solution for their integration ( Council of Europe Report, 2022). In Greek islands, the first arrival and asylum process facilities in Lesvos, Chios, Samos, Leros, and Kos are often criticised for their inhospitability and inhumane reception conditions. The Closed Controlled Access Centers (CCACs), also known as Multi-Purpose Reception and Identification Centers (MPRICs) display barbed wires and video surveillance that gives them detention-like appearance. Italy also infamously developed pre-removal detention centers for soon to be deported migrants, such as Centri di Permanenza per i Rimpatri (accommodation centers for repatriation) and ex-Centri di Identificazione e Espulsione (identification and expulsion centers) in Rome, Turin, Caltanissetta, and Brindisi ( Esposito *et al.*, 2019). Airports and places of entry can also sort migrants under the Asylum Procedures Directive.

(2) After a stay in closed center where incomers are sorted and selected according to their asylum-proof profile, the “dublinable” incomers are transferred to open centers or accommodation centers (asylum processing centers) where they can start their asylum procedure, as in the Italian Centri di accoglienza per richiedenti asilo, or CARAs. Created under Law no. 189/2002 and implemented in 2004, these ‘open centers’ allow asylum seekers out during daytime with notice to the center’s director and authorities ( Campesi, 2015).

(3) Integration (or specialized) centers represent the last stage before an independent integration into the welcoming society ( EASO, 2021). These are particularly fit for families and unaccompanied minors, yet they are insufficient in numbers and capacity as “the average length of stay for people seeking asylum subject to Dublin procedures was 59 to 73 days in 2021” ( Women’s Refugee Commission, 2022). Different types of initiatives have developed across Europe. The Switzerland’s federal asylum centers combine welcoming, asylum-seekers’ application support and integration, with legal and social support. In Luleå, Sweden, 25% of vacant flats are expected to be made available for refugees (families and minors in priority). In Marseille, France, and in the Swedish region Norrbotten, ‘sliding lease’ systems have been implemented, i.e. sublet accommodation rented by an NGO or social services. The lease is then transferred to the migrant once independent. Accommodation being key in the integration of migrants in the receiving societies, German NGO Kontakstelle Wohnen assists refugees in their search of accommodation.

Beyond indoor spaces of reception or transit for migrants, there are such outdoor spaces as the Calais infamous “jungle”, an outdoor space for migrants and threshold to inhumanity.

### Women migrants’ reception

Today, migrant women still face dangers on the migration journey (trafficking, GBV and health-related risks). Mixed reception centers are regularly accused of not offering safety nor adequate sanitary conditions whereas reception facilities are key to women migrants’ access to healthcare, education and employment ( Phillips, 2006), but the lives of women in pre-removal detention centers is shaped by intersectional forces such as gender, race and sexuality as women inmates are at higher risks of GBV.

Right after the Russian invasion in Ukraine, global solidarity led to the development of private housing for Ukrainian women refugees ( EUAA Private Accommodation, 2022a), with the adverse risk and consequence of exploitation and harassment ( Council of Europe Report, 2022). Decision (EU) 2022/382 reactivated the Council Directive 2001/55/EC on Temporary Protection (TPD) to accommodate and ease the integration of large influx of Ukrainian refugees in the EU, yet the FEANTSA report reveals an over-relying on private accommodation. In Lublin, Poland, Ukrainian refugees were matched with hosts in private accommodation, and the initiative was managed by local civil societies ( Council of Europe Report, 2022). Individual housing has been successfully tried outside the EU, for instance in Canada with the Syrian Refugee Initiative, with such benefits as greater integration success and lower costs for the State.

In France, although centers for women exist, they are rare so the women are often sheltered in hotels and tentatively separated from male newcomers when possible. The Centre d'Accueil pour Demandeurs d'Asile (CADA) hosts asylum seekers and, when too overcrowded, the Hébergement d'Urgence pour Demandeurs d'Asile (HUDA) can take over. These structures are only meant for asylum seekers about to be “dublined”. When institutionalized migrants’ reception is unavailable, homeless shelters can be the only option available to newcomers, hence inappropriately mixing two societal realities into one: homelessness and migration. Adding to the detention center system, migration seems to be assimilated to either homelessness or criminality in this context.

Other types of reception centers exist such as semi-closed centers as the Ödos center (Spain) that allows women migrants to leave the center yet keeps it closed and away from the city to protect them. In Germany, semi-closed centers allow mobility limited to 48h and to the surroundings of the center. These are meant to secure reception and asylum application conditions. In initial centers, access to employment is generally not possible, so as not to impact the asylum application process. The length of stay varies between 3 (Ödos: time for DNA results) to 24 months (AnkER center) ( Women’s Refugee Commission, 2022). In the final integration stage, the SURT NGO in Spain assists non-EU women migrants in their labour market insertion ( Council of Europe Report, 2022). In Poland, asylum-seekers can be funded in private accommodations to ease their integration, yet the 180€/month allowance is insufficient to meet migrants’ needs ( FRA, 2019).

## Challenges identified by WEMov

The questions raised by this brief are:

• How to avoid detaining women migrants in prison-like centers?

• Why are reception centers needed for women?

• How can reception centers become empowerment tools for women migrants?

The key issues identified by WEMov concern:

1. Dignified reception for women migrants

2. Safety in women’s reception centers

3. Mental and physical health

4. Integration support: employment and accommodation

5. Harmonisation of practices across EU countries

6. A networking system of information

### Dignified reception for women migrants

The infamous Jungle of Calais is exemplary of what awaits women migrants in similar places, the number of women rising from 2009 to 2016 in the “Jungle” and surrounding squats. In 2016, women and girls represented 14% of the migrant population in the area. Gynécologie Sans Frontières reports the lack of adequate reproductive and basic healthcare as well as sexual violence against women causing pregnancy and mental and physical harm. Similarly, Médecins Sans Frontières notes the inadequate reception conditions for pregnant women migrants in Greece. Inadequate housing is shown in the lack of sanitary facilities with for instance 6 showers for 600 people in Voenna Rampa, Bulgaria. In Calabria, Italy, no bathrooms at all were offered to the 500 container residents ( Bekyol *et al.*, 2016).

### Focus on safety in women’s reception centers

In a 2020 brief on Gender and Migration, the European Institute for Gender Equality acknowledged that women and children were at greater risk of GBV and hence are in greater need for protection ( EIGE, 2020). A 2022 Council of Europe report further noted that mass reception accommodation was often inadequate to their needs in healthcare, education and housing ( Council of Europe Report, 2022).

Yet, according to Human Rights Watch, in Greece women asylum-seekers are often confined for months with unrelated men sharing facilities with close to no-intimacy, with consequential risks of sexual abuse. For instance, Maha from Iraq reported being held in a cell with 60 men for two weeks, which led to suicidal thoughts. Fatima from Algeria was the only woman in a cell with single men for 20 days and was under extreme stress when using the sanitary facilities. Suha from Morocco recalls there being 2 girls with 60 or 70 men in the Fylakio pre-removal center ( Human Rights Watch, 2018). These testimonies (and more on Info Migrants, 2018) call for immediate ending of such practices and implementation of national and European guidelines requiring to separate men and women, unless related, in reception facilities. Such situations have also been condemned by Amnesty International ( Human Rights Watch, 2018).

In Belgium, Article 83 of the Reception Act states that men and women should have separate sanitary and dormitory facilities and privacy ensured. Furthermore, male staff and guards can lead to uncomfortable situations for women and couples. Migrant women do not feel safe in detention centers and open facilities dominated by men, where mixed corridors and common sanitary facilities increase the feeling of insecurity ( Nederlandstalige Vrouwenraad, 2010).

The Women’s Refugee Commission (WRC) conducted research in the Greek Moria center where women and girls described complete lack of protection from sexual assaults and risks of sexual exploitation. Hence, women and children tend to stay confined in tents for safety, and the same was reported in France’s Calais or Greece’s Chios and Samos. Sanitary facilities that are not separate for men and women are places of insecurity for women and girls.. Nahla from Aleppo, Syria: “This camp is not safe. I won’t even go to the toilet by myself. […] We don’t want a lot. We just want a safe place, to have a normal life again”.

### Mental and physical health

According to Article 17(2) of Directive 2003/9/EC, medical and psychological care must be provided for by EU Member States welcoming migrants: “Member States shall ensure that material reception conditions provide an adequate standard of living for applicants, which guarantees their subsistence and protects their physical and mental health”. The necessity for separate dormitories that can be locked as well as health and psychological care by trained staff are underlined as key to the reception of vulnerable migrant women.

Inadequate housing and sanitary facilities can indeed cause stress and trauma, a Council of Europe report denounces ( Bekyol *et al.*, 2016). Yet, detention centers generate anxiety among inmates because of freedom and privacy deprivation which lead to serious mental health issues and trauma. Médecins du Monde reports that a lack of privacy can severely impact asylum seekers’ psychological health. The poor infrastructures and remoteness are an issue to access social services and integrate the host community. Additionally, social and medical staff are not always adequately trained to address the needs of migrants who have gone through extreme violence. The shortage of interpreters can also lead to inadequate handling and thus mental health vulnerability, and police authorities are often insufficiently trained to GBV and violence survivors. ( Nederlandstalige Vrouwenraad, 2010).

### Integration support: employment and accommodation

A 2022 Council of Europe report highlights women migrants’ resilience, and urges the local communities to make use of their potential ( Council of Europe Report, 2022). Noteworthy, vocational training was introduced as a necessity in Article 12 of Directive 2003/9/EC as a possibility that EU Member States may offer migrants. Employment is necessary for the migrant women to succeed in integrating, yet this is only possible with language skills and education, that must be part of the reception process but is yet impeded by deskilling and discrimination in highly competitive labour markets.

Beyond cultural differences and their role as mothers (single parents are most Ukrainian refugees for instance) with sometimes young children, women migrants must also find ways to move and access to public transports can represent employability obstacles. Hence, not only childcare but also eased mobility to access employment contribute to successful integration.

### Harmonisation of practices across EU countries

The Reception Conditions Directive (2013/33/EU) focuses on harmonizing practices across the EU to meet the needs of vulnerable migrants. The 2015 European Agenda on Migration (IP/15/4956) also introduced management standards meant to harmonize practices across the EU ( EASO, 2021). Yet, states like Bulgaria and Malta host migrants in overcrowded reception centers with insufficient integration support. The detention duration also varies among Member States between 1 to 20 months, with remarkable impact on mental health.

The need to harmonize practices is clear in the definition of migrants’ vulnerability across EU Member States. Indeed, Articles 21 and 22 of Directive 2013/33/EU guarantee adequate housing for vulnerable migrants. Bulgaria, for instance, limits vulnerability to unaccompanied minors, single parents, pregnant women, the disabled, the elderly and survivors of abuse. Belgium extends its definition to survivors of human trafficking, while Slovakia rather defines people with specific needs such as pregnant women, the disabled and survivors of tortures. In Austria, definition variations are regional rather than national. These introduce national and European distinctions that impact migrants’ reception and integration across the EU.

Although Directive 2013/33/EU was a step towards the harmonisation of practices, efforts still need to be made to better address migrants’ vulnerability across the EU. For instance, Belgium and Germany, have opened dedicated centres for families, women and vulnerable migrants, and Poland, Hungary, Romania and Slovakia have specific accommodation for single women ( Council of Europe Report, 2022).

### A networking system of information

An information networking system is missing between reception centers across Europe and neighbouring countries to document women migrants’ profiles and needs from one center and country to the next. Although the EU police forces can rely on the Eurodac database, reception centers and NGOs do not have access to a centralized system of information. Gender-disaggregated data is key to better inform the local communities of the demographic structure of the newcomers, better understand women’s migration, address situations of vulnerability and improve the reception of women migrants ( Council of Europe Report, 2022). Better coordination with local civil societies is also regularly advertised in reports and directives, a central information network system would also benefit both international organisations and migrants. In its 2010 report, Nederlandstalige Vrouwenraad requests the EU member states to share data on vulnerable migrants, including pregnant women and victims of sexual abuse.

## WEMov’s approach

WEMov’s research stems from visits in 3 reception centers (one in the EU and two outside the EU) and all three stand as models that complement each other. Kutchinate, a day center in Israel, was the first facility visited by WEMov and its key features are:

• Kutchinate offers crafting classes to turn migrants into workers with economic and professional benefits. Migrants create baskets sold in Kutchinate’s shop. They learn accounting and this increases their autonomy and integration;

• Kutchinate also offers administrative, psychosocial and recreational support to the women migrants who are welcome to socialize in the day center;

• The center is located in central Tel Aviv, hence easily accessible in public transport for the women migrants.

Its main flaw is that it is only a day center that no longer offers accommodation to the women migrants, who struggle to find suitable and decent accommodation in Tel Aviv, accommodation being central to women migrants’ well-being and integration

The second facility WEMov has visited is the Sarajevo Temporary Residence Center (TRC) Usivak in Bosnia & Herzegovina, which particularity is the presence of intergovernmental organisations such as UNICEF, UNHCR and IOM, among others, hence ensuring adequate reception conditions for the migrants. Its main strengths are:

• IOM Bosnia’s No Nation Fashion initiative allows women migrants to make fashion, with an international fashion show, exemplary of the Global Compact for Migration (UN Department of Economic and Social Affairs);

• With separate quarters, the center separates families/women/unaccompanied minors from men on the move in order to guarantee their safety;

• It also offers distinct educational and recreational activities for children;

• Beautiful, the centre is colourful with posters and paintings on its buildings set in the middle of beautiful nature.

Its only disadvantage is its remoteness as well as its containers’ structure, that may be unwelcoming to some, but it is actually countered by the colourful displays across the center.

The last reception center that WEMov has visited is led by Programa Ödos in Southern Spain which can welcome about 40 women migrants and children and is structured in two parts: the main first arrival center, and the related integration structure for asylum-seekers. It displays:

• Cooperation with the local community with the monitoring of the local police, as well as quality clothing, equipment and toys donated by the locals;

• The asylum seekers’ facility is set in the town centre, easing the women and children’s integration in the local community;

• The staff (social workers and psychologists) includes men and women trained for GBV survivors. This mixed gender staff approach is key for the women to progressively regain trust in men, despite their GBV past experiences;

• Attending the local school, children live a “normal” life in the local community;

• The place is beautiful, clean and healthy with adapted facilities: a canteen, common kitchen, a library, classrooms, a swimming pool and sports pitch;

• Each woman is given a room for herself and children and the women rotate cleaning shifts of the shared facilities.

The only restriction the center seems to have is its remoteness from towns, which protects women and gives them space to build themselves up after traumatic experiences, yet may also induce boredom and a sense of isolation. Nevertheless, the second facility for asylum seekers is set in the center of town, which eases the integration of the women in the local community.

WEMov believes that these are model facilities that should be replicated and followed. Observation visits to these centers should be organized to improve other EU reception centers and promote best practices.

## WEMov’s recommendations

In light of the above study, WEMov recommends the following provisions and improvements:

### Short-term improvements in reception

***Gender mainstreaming in migrants’ reception***

1) Gender mainstreaming must be introduced in mass mixed reception centers;

2) WEMov recommends identifying women migrants’ vulnerability in first reception centers by resorting to intersectional tools ( Council of Europe Report, 2022);

3) In reception and transit centers, there should be dedicated spaces for victims of GBV;

4) LGBTQIA+ migrants are generally absent from policies and directives, yet their needs must be accommodated upon reception with specific trained staff and separate facilities.

***Staff***

5) Reception center’s staff should be trained in gendered aspects of migration (GBV; family law; psychology; labour integration for women);

6) WEMov recommends training the police and authorities to improve women’s reception;

7) WEMov stresses the importance to have both female and male staff in reception centers for women to progressively build trust in men yet ensure women’s safety;

8) In case of assault in a mixed center, the women should be able to get legal and psychological support from trained and dedicated staff;

9) Migrant women should be able to complain about reception centers’ staff. For instance, in Belgium and Germany, complaint management systems have been introduced ( Bekyol *et al.*, 2016);

10) The staff should be able to speak the language of the migrant women (or translators introduced during interviews), and they should be trained in intercultural issues.

***Reception centers’ structure***

11) The Sarajevo TRC Usivak has a linear structure, which is not as welcoming as the hacienda-like architecture of the Ödos center with a central yard for children to play and women to gather. A modular approach, circular with a nerve for easy flow is recommended by the EU Agency for Asylum (2022), yet reminiscent of a panopticon;

12) WEMov recommends not isolating reception centers to ease migrant women’s integration, yet safety should be guaranteed with the local police’s cooperation.

***Focus on safety and health***

13) In collective reception centres, small size accommodation should be the rule to avoid risks of violence but also to allow the staff to work more efficiently with reduced control of people and space. Ödos only accommodates 40 people maximum and thus the migrants have space and quiet to prepare their integration;

14) Behishta from the Sarajevo Temporary Residence Center (TRC) Usivak described the kindness and care of the staff as well as the activities provided in the camp, which allowed her recovery from depression, and the same was observed at Ödos;

15) Social services and dedicated psychologists should be present in all reception centers;

16) Self-defense and protection rights should be taught to women in reception centers;

17) Reception centers must be places of empowerment and not detention centers. Sports and diverse empowering recreational and cultural activities should be introduced in all reception centers for therapeutical, integrative and skill-mining purposes;

18) Animals and pets can also have therapeutic effects on women and children.

### Changes in practices

19) There should be more specialized reception centers. If separate accommodation is not possible, lockable rooms and separate sanitary facilities must be provided;

20) WEMov recommends to introduce privacy policies across the EU, for all migrants, but especially women and girls as well as unaccompanied minors;

21) Monitored private housing programs should be developed, yet the risks of exploitation, and safety of the newcomers should be controlled;

22) WEMov recommends using unoccupied accommodation to host migrant women, families and children, as it was tried in Luleå, Sweden, and also develop such schemes are the ‘sliding lease’ system tried in Marseille, France, for women migrants’ autonomy;

23) Civil societies must act as key actors in the migrants’ reception and integration;

24) Women migrants must be included in the local community for their empowerment;

25) The Warsaw reception center has a free “shop” for residents.

26) Reciprocal observation visits between EU reception centers can foster best practices. Ödos is a model: clean and welcoming, with recreational and educational activities and excellent relationships with the local community and authorities;

27) Practices should be harmonized across the EU and data more transparent on reception centers with greater coordination and implementation of best practices;

28) Reception centers should be regularly visited by EU officials, checked and assessed to ensure the abidance of EU regulations and reception directives.

### Strategic changes

29) WEMov estimates that the reception contingency should be (at least) tripled in all EU Member States to allow for decent reception conditions;

30) WEMov recommends to plan the integration of migrants in keeping with the EU’s Action Plan on Integration and Inclusion (2021–2027) with the cooperation of local communities and workers in the search for accommodation and employment;

31) WEMov recommends introducing a European tracing system ( Bekyol *et al.*, 2016) to improve migrants’ reception by tracing their needs (not to police them as Eurodac) within the EU and neighbouring transit states. A tool referencing the migrants’ profiles and medical record should be developed for reception centers across the EU and neighbouring transit countries for better assistance and reception;

32) Gender disaggregated data are key to better understand, plan and address the needs of women migrants, they should be made available across the EU.

### Changing the approach to migration

33) WEMov recommends that the EU challenges the overwhelming burden-led discourse on migration by stressing the EU’s multiethnic and multicultural essence. For instance, watery analogies should be avoided and figures checked. If EU institutions change the macro-narrative on migration, xenophobic attitudes towards newcomers will decrease.

34) The EU should nuance the vulnerability trope that frames migrant women as burdens and thus impacts their integration into receiving nations. It should emphasize their skills and resources, as resilient and empowered actresses whose strengths can be useful.

35) The focus on asylum should be countered and reception for non-asylum seekers developed;

36) Specialized facilities for migrants leaving poverty, betterment migrants and GBV survivors should be opened with specific support suited to these profiles;

37) Migration not being a crime, migrants’ detention centers should not exist. Currently, WEMov recommends to urgently limit length of stay in detention centers to 2 weeks maximum by introducing more staff and efficient system of reception;

38) Reception centers should not criminalise migration by confining newcomers.

## Conclusion

There is an overwhelming focus on asylum-seekers in reports on reception centers. Yet, this is misleading and prevents a comprehensive understanding of the variety of migration patterns and profiles. WEMov believes that destitution should be considered a case for protection and the reception of poverty-stricken migrants should also be organized at EU level, studies showing that women are often specifically affected by poverty as traditionally responsible for feeding the family. Hence, WEMov considers the literature on reception centers and policies flawed and incomplete as not all migrants’ profiles are included and non-persecution types of vulnerabilities excluded. Isn’t betterment migration a valid reason for migration worthy of protection?

## Ethics and consent statement

Ethical approval and consent were not required.

## Data Availability

No data are associated with this article
