# Supplementary material for: Reception Centers and Practices for Women Migrants
Source: Open Res Eur. 2025 Jun 12;4:199. Originally published 2024 Sep 9. [Version 2] doi: 10.12688/openreseurope.18207.2 (PMC12238843; doi:10.12688/openreseurope.18207.2)
Supplement: Peer review history

# Reviewer report: Reviewer response for version 2

**DOI:** 10.21956/openreseurope.22035.r55635

**Published:** 2025-08-12

**Authors:** Naïké Garny

**Affiliations:** 1 KU Leuven, Leuven, Leuven, Belgium

The article, presented as an open letter by Marie Ruiz and Stellamarina Donato, critically examines the reception of women migrants through a gender-mainstreaming lens. Drawing on research from COST Action *Women on the Move*(CA19112) and field visits to reception centers in Israel, Spain, and Bosnia & Herzegovina, the authors analyze the adequacy and diversity of reception facilities across Europe. They explore how international and European frameworks—particularly UN and EU policies—are implemented on the ground, with a focus on women’s safety, well-being, and integration.

 The article highlights persistent shortcomings in meeting the specific needs of women, especially those at risk of gender-based violence, and critiques the carceral conditions found in some centers. It also identifies promising practices and operational challenges observed across various sites. In response, the authors offer a series of concrete recommendations at both policy and operational levels. These include the adoption of gender-sensitive approaches, improved staff training, greater access to specialized support services (such as medical, psychological, and vocational aid), and the promotion of safe, inclusive environments that foster autonomy and community inclusion for women migrants.

 The following points are suggested for further consideration:

**1. Introduction**

- The acronym *WEMov* should be explained the first time it is mentioned.
- It would be helpful to indicate when the research was conducted and how long the fieldwork lasted.

**2. The Three Reception Phases**

- Is the Calais Jungle considered part of the three phases? Its mention at the end of the section feels abrupt, especially if the focus is primarily on official centers.
- Additionally, it would be valuable to address other types of shelters and facilities found across Europe. For example: collective shelters, NGO-run centers, and squats. Since you include informal outdoor spaces like the Calais Jungle alongside official centers, it would be interesting to also present the broader spectrum of indoor alternatives.
- The use of the term *“dublinable”* may need clarification. As I understand it, a dublinable person is one who must be transferred back to the first EU country where they registered (i.e., where their fingerprints were first taken). However, your explanation suggests it has a different meaning here, especially if such individuals are later transferred to open centers.

**3. Women’s Migrant Reception**

- When mentioning *CADA* and *HUDA*, it would be helpful to clarify whether these facilities also accommodate women asylum seekers. If so, please make this more explicit.
- It is not always clear whether you are referring specifically to asylum seekers or to migrants in general in this section. If you are addressing both, it may be useful to distinguish between them more clearly, as they face different realities.

**4. Dignified Reception for Women Migrants**

- The discussion becomes somewhat confusing here due to the fluid movement between encampments, squats, and formal centers. Clarifying which type of reception setting you are referring to at each point would improve the reader's understanding.

**5. WEMov’s Approach**

- The phrase *“to turn migrants into workers”* can come across as objectifying, as it implies a lack of agency on the part of migrants. Consider rephrasing to reflect their autonomy and role in the process.

**6. Changes in Practices**

- Point 25 reads more like a factual statement than a recommendation, which shifts the tone of the section. Rephrasing it as a recommendation would align better with the overall structure.

**7. Conclusion**

- The conclusion feels somewhat disconnected from the rest of the brief. While the topic of migrant betterment is mentioned earlier, its strong emphasis here seems disproportionate—especially since the brief focuses on reception conditions for women migrants. I would suggest revisiting this section and ensuring the conclusion aligns closely with the core subject.
- More broadly, the terms *migrants* and *asylum seekers* are used somewhat interchangeably throughout the text. It would be helpful to specify which group you are referring to in each context, and to clarify the type of reception centers being discussed. This would help avoid confusion when shifting between different geographic locations or facility types. Even though your recommendations may apply broadly, this added precision would improve clarity.

**Overall Impression:**

 This is a highly valuable and necessary brief. The recommendations are clear and well thought out. I especially appreciate the incorporation of an intersectional perspective—it is essential in this context.

Where applicable, are recommendations and next steps explained clearly for others to follow? (Please consider whether others in the research community would be able to implement guidelines or recommendations and/or constructively engage in the debate)

Yes

Does the article adequately reference differing views and opinions?

Yes

Are all factual statements correct, and are statements and arguments made adequately supported by citations?

Yes

Is the rationale for the Open Letter provided in sufficient detail? (Please consider whether existing challenges in the field are outlined clearly and whether the purpose of the letter is explained)

Yes

Is the Open Letter written in accessible language? (Please consider whether all subject-specific terms, concepts and abbreviations are explained)

Yes

Reviewer Expertise:

Women's resistance, reception practices, women migration in Belgium, feminist and intersectional perspectives of research

I confirm that I have read this submission and believe that I have an appropriate level of expertise to confirm that it is of an acceptable scientific standard.

# Reviewer report: Reviewer response for version 2

**DOI:** 10.21956/openreseurope.22035.r55437

**Published:** 2025-07-08

**Authors:** Nicholas Harney

**Affiliations:** 1 Western University, Western University, Canada

Thanks. These revisions are satisfactory.

Where applicable, are recommendations and next steps explained clearly for others to follow? (Please consider whether others in the research community would be able to implement guidelines or recommendations and/or constructively engage in the debate)

Partly

Does the article adequately reference differing views and opinions?

Partly

Are all factual statements correct, and are statements and arguments made adequately supported by citations?

Partly

Is the rationale for the Open Letter provided in sufficient detail? (Please consider whether existing challenges in the field are outlined clearly and whether the purpose of the letter is explained)

Partly

Is the Open Letter written in accessible language? (Please consider whether all subject-specific terms, concepts and abbreviations are explained)

Yes

Reviewer Expertise:

migration and urban life

I confirm that I have read this submission and believe that I have an appropriate level of expertise to confirm that it is of an acceptable scientific standard.

# Reviewer report: Reviewer response for version 2

**DOI:** 10.21956/openreseurope.22035.r55436

**Published:** 2025-06-16

**Authors:** Elena Rubini

**Affiliations:** 1 CRIMEDIM - Center for Research and Training in Disaster Medicine, Humanitarian Aid and Global Health, University of Eastern Piedmont, Vercelli, Piedmont, Italy

Thank you for amending the manuscript following my comments. I have no additional points to raise.

Where applicable, are recommendations and next steps explained clearly for others to follow? (Please consider whether others in the research community would be able to implement guidelines or recommendations and/or constructively engage in the debate)

Yes

Does the article adequately reference differing views and opinions?

Partly

Are all factual statements correct, and are statements and arguments made adequately supported by citations?

Partly

Is the rationale for the Open Letter provided in sufficient detail? (Please consider whether existing challenges in the field are outlined clearly and whether the purpose of the letter is explained)

Yes

Is the Open Letter written in accessible language? (Please consider whether all subject-specific terms, concepts and abbreviations are explained)

Partly

Reviewer Expertise:

Global health, migrants' health, GBV, SRHR, equity in access to care

I confirm that I have read this submission and believe that I have an appropriate level of expertise to confirm that it is of an acceptable scientific standard.

# Reviewer report: Reviewer response for version 1

**DOI:** 10.21956/openreseurope.19677.r45271

**Published:** 2024-10-25

**Authors:** Elena Rubini

**Affiliations:** 1 CRIMEDIM - Center for Research and Training in Disaster Medicine, Humanitarian Aid and Global Health, University of Eastern Piedmont, Vercelli, Piedmont, Italy

The article summarizes the international  as well as European frameworks for migrants' reception, the different types of reception centers, as well as the challenges identified by the authors after visiting three reception centers (in Israel, Spain, and Bosnia & Herzegovina). The authors then suggest a series of recommendations at policy and operational level. 

 The article focuses specifically on women migrants and the challenges they face in reception centers, highlighting criticalities and best practices. 

 - I would suggest authors to revise some of the English language they employ, as at times it becomes too informal (ex. "being run").

 - I believe some of the quotes should become more smooth (e.g., "According to the UNHCR... 60% of refugees in the EU)

 - The Universal Declaration of Human Rights does not "guarantee" but "calls for". Also SDGs do not guarantee, but highlight the need for ensuring the safety and well being of migrants. 

 - CEDAW: first write the explanation, then in parentheses add the acronym

 - CEDAW recommendations apply and suggest (without s)

 - add "e.g.," before "housing, health, food, clothing and education"

 - Strategy on gender equality with capital S

 - I believe that the statements reported in the paragraph on the New Pact on Migration and Asylum should be more nuanced since there might be a discrepancy between the policies and the reality of lives for migrants (e.g., smuggling and trafficking). Moreover, I believe authors should also add a paragraph on possible issues connected to these policies in terms of fulfillment of the human rights of migrants (e.g., database to fingerprint monitor non-EU migrants, smuggling and trafficking, use of AI). 

 - I believe that the gender and migrant components of the identity of the population taken into consideration should be also analyzed in an intersectional perspective as reinforcing the challenges faced by them. Add a brief sentence on this.

 - The paragraph "Typologies of reception centers" does not describe them, but is in reality a brief historical overview on women travelling. I do not believe the parallel between inns and reception centers to be correct. Moreover, the description of the different types of reception centers is provided in the next paragraph. I suggest removing the paragraph "Typologies of reception centers" and incorporating the reflections on the issues encountered by women. Also, I would not limit those to health and safety, but would add an explicit reference to gender based violence.  

 - Dubliné-e is a term used in France. I would add an explanation of what "Dublinable" means for non French speaking readers. 

 - Please, add a reference for the part on the reception management in Greek islands. 

 - Paragraph "Women migrants' reception": please see some literature on the continuum of violence in migrant women, as they do not face the threat of GBV only in their home country and in transit, but also in the host country. 

 - In the same paragraph, the authors use "are accused" which combined with the "whereas reception facilities are key to women migrants.." gives the idea that you do not share this view. I believe this part should be rephrased in a more nuanced and objective manner. 

 - The lives ARE shaped

 - What is the "Jungle of Calais"? Please explain to non French readers. 

 - Please add references for Gynécologie Sans Frontières and Médecins sans Frontiers.

 - I would avoid putting even ficticious names (Maha, Fatina, Suha, Nahla) and replace them with a woman, a woman migrant.

 - Please add a reference for the paragraph from WRC and for the quote from Nahla from Aleppo.

 - "Inadequate housing and sanitary facilities can indeed cause stress and trauma, a Council of Europe report denounces" this quote seems more journalistic in nature. Please rephrase it to be more scientific.

 - Please add a reference for Médecins du Monde. 

 - Employment is necessary for migrant women (without THE).

 - Change into "Most Ukrainian refugees are single mothers"

 - "to turn migrants into workers": to provide migrants with the skills to become

 - "to make fashion": to tailor clothes

 - "quarters": sections

 - "Beautiful, the centre is colourful with posters and paintings on its buildings set in the middle of beautiful nature." : this statement is too subjective. I suggest the following: "The walls of the centre, itself built in the middle of nature, are decorated with paintings".

 - "but it is actually countered by the colourful displays" would delete.

 - "trained for GBV survivors": trained to support GBV survivors

 - "This mixed gender staff approach is key for the women to progressively regain trust in men, despite their GBV past experiences" is this the opinion of the staff center? If so I would add that "the staff reported this mixed gender...". Otherwise I would add references to evidence in the literature on this. 

 - live a “normal” life: are integrated in 

 - "for herself and HER children"

 - "LGBTQIA+ migrants are generally absent from policies and directives, yet their needs must be accommodated upon reception with specific trained staff and separate facilities" please add some considerations on LGBTQIA+ migrants in your Introduction. 

 - "WEMov recommends training the police and authorities to improve women’s reception" Please add considerations on the current level of preparedness of police and authorities in the introduction. 

 - "assault" if it is referred to sexual ones I would add sexual.

 - to FORMALLY complain

 - (or translators introduced during interviews),: or cultural mediators should be present

 - panopticon: please explain this or change into "prison". 

 - "Behishta from the Sarajevo Temporary Residence Center (TRC) Usivak described the kindness and care of the staff as well as the activities provided in the camp, which allowed her recovery from depression, and the same was observed at Ödos": rephrase to "the activities and support provided in the camp can positively affect the stay of migrants and their mental health".

 - "Self-defense and protection rights should be taught to women in reception centers": Women migrants should be made aware of the different forms of GBV and understand how to face it and formally complain about it.

 - "Animals and pets can also have therapeutic effects on women and children" either mention it before providing evidence or delete.

 - " The Warsaw reception center has a free “shop” for residents." : seems a bit out of the blue.

 - Changing the approach to migration: please integrate these considerations also in the Introduction.

 -  "for migrants leaving poverty, betterment migrants and GBV survivors" : to improve the condition of migrants regarding their socioeconomic status and their exposure to GBV

Where applicable, are recommendations and next steps explained clearly for others to follow? (Please consider whether others in the research community would be able to implement guidelines or recommendations and/or constructively engage in the debate)

Yes

Does the article adequately reference differing views and opinions?

Partly

Are all factual statements correct, and are statements and arguments made adequately supported by citations?

Partly

Is the rationale for the Open Letter provided in sufficient detail? (Please consider whether existing challenges in the field are outlined clearly and whether the purpose of the letter is explained)

Yes

Is the Open Letter written in accessible language? (Please consider whether all subject-specific terms, concepts and abbreviations are explained)

Partly

Reviewer Expertise:

Global health, migrants' health, GBV, SRHR, equity in access to care

I confirm that I have read this submission and believe that I have an appropriate level of expertise to confirm that it is of an acceptable scientific standard, however I have significant reservations, as outlined above.

# Response

**Published:** 2025-05-02

**Authors:** Marie-José Ruiz

**Affiliations:** Anglophone Studies, Universite de Picardie Jules Verne, Amiens, Hauts-de-France, France

We thank the reviewer for their close reading and detailed feedback, which we found insightful and valuable. We appreciate the overall recognition of our contribution and welcome the opportunity to clarify, refine, and strengthen the manuscript in the light of your suggestions. Below is our response to each point raised. **Language and Style**

- **Informal Language**: We appreciate the feedback on expressions and academic phrasing throughout the text.
- **Smoother Integration of Quotes**: We have have taken into account advice for better flow and more fluidly into the narrative.
- **CEDAW**: We understand the suggestion to spell out the full name first before introducing the acronym.
- **Other Edits**: We have adopted recommendations for minor stylistic changes to improve clarity and consistency.

 
**Policy Analysis and Context**

- **Nuancing EU Policies**: We believe it is necessary to acknowledge the gap between official EU policies (e.g., the New Pact on Migration and Asylum) and on-the-ground realities, particularly regarding human rights concerns around surveillance, AI, and biometric data collection.
- **Intersectionality**: it is indeed key to recognize how gender, migration status, race, and other factors compound vulnerabilities.

 
**Structural Suggestions and Clarifications**

- **Typologies of Reception Centers**: We believe the paragraph labeled “Typologies of Reception Centers” was more historical than classificatory and was necessary to set the work in the longue durée.
- **Dublinable**: the term is being used in other contexts, so we have kept it, as we believe it is clear enough
- **Greek Islands**: necessary reference to support statements on reception conditions in Greek island centers.

 
**Content Accuracy and Tone**

- **Continuum of GBV**: There is indeed a continuum of violence experienced by migrant women—not only in origin and transit countries, but also in host countries.
- **Subjective Language**: Some statements can be more objective as suggested.
- **Names in Testimonies**: Individual names are referenced in our publications following legal agreement and are accessible in videos on our website, so there is no need for anonymation.

  We believe that the manuscript reflects a clear structure, and nuanced policy critique. We are grateful for your attentive and thorough review. **Marie Ruiz & Stellamarina Donato**

# Reviewer report: Reviewer response for version 1

**DOI:** 10.21956/openreseurope.19677.r44430

**Published:** 2024-10-15

**Authors:** Nicholas Harney

**Affiliations:** 1 Western University, Western University, Canada

The article, or 'Open Letter' authored by Marie Ruiz and Stellamarina Donato, explores the adequacy of reception centers for women migrants from a gender-mainstreaming perspective. It focuses on practices in European reception centers, examining their impact on women's safety, integration, and overall well-being. The authors utilize research from COST Action Women on the Move (CA19112) to highlight the variety of reception facilities and their alignment with the specific needs of women migrants. The paper discusses international frameworks, including the UN and EU policies, and emphasizes that improvements are necessary in the reception systems to better support women, especially those vulnerable to gender-based violence (GBV). The article also critiques detention-like conditions in some centers and advocates for best practices to be shared and implemented across Europe.

 Key recommendations include enhancing staff training, integrating gender-specific provisions, ensuring safety and privacy, and fostering community integration. The authors call for gender-sensitive approaches and emphasize the need for specialized support services, including medical, psychological, and vocational assistance. The open letter could be improved in these four areas below:

- **Incorporation of Empirical Data**: The authors need to supplement their observations with more robust, quantifiable data from a larger sample of reception centers. This would include statistics on GBV incidents, health outcomes, and the overall success rates of integration programs for women migrants.
- **Detailed Methodology**: The methodology section must be expanded to describe the research design, sampling methods, and data analysis techniques. What was it? In addition to the sweeping context, we are told that 3 centres were observed but how? This would allow readers to understand how the conclusions were reached and to assess the reliability of the findings.
  The methodology section lacks detail, especially regarding how data were collected in the case studies. Although the authors visited three centers, the criteria for selecting these centers, the scope of interviews, and the data analysis process are unclear.
   The authors should expand the methodology section by providing specific details on their field research methods. They should clarify how they selected the centers, how many interviews were conducted, and whether the interviews followed a structured or semi-structured format. Additionally, a section on data analysis (e.g., thematic coding) would enhance transparency.
- **Feasibility of Recommendations**: Some of the recommendations should include more practical details on how they can be implemented, including examples of best practices from other regions. More information on the financial and logistical feasibility of these recommendations is needed for policymakers to act on them.
- **Critique of Policy Enforcement**: While the article references many international and EU policies - too many acronyms (!) -  it should include a critical assessment of how well these policies are enforced and the gaps that exist between theory and practice.

Where applicable, are recommendations and next steps explained clearly for others to follow? (Please consider whether others in the research community would be able to implement guidelines or recommendations and/or constructively engage in the debate)

Partly

Does the article adequately reference differing views and opinions?

Partly

Are all factual statements correct, and are statements and arguments made adequately supported by citations?

Partly

Is the rationale for the Open Letter provided in sufficient detail? (Please consider whether existing challenges in the field are outlined clearly and whether the purpose of the letter is explained)

Partly

Is the Open Letter written in accessible language? (Please consider whether all subject-specific terms, concepts and abbreviations are explained)

Yes

Reviewer Expertise:

migration and urban life

I confirm that I have read this submission and believe that I have an appropriate level of expertise to confirm that it is of an acceptable scientific standard, however I have significant reservations, as outlined above.

# Response

**Published:** 2025-05-02

**Authors:** Marie-José Ruiz

**Affiliations:** Anglophone Studies, Universite de Picardie Jules Verne, Amiens, Hauts-de-France, France

We thank the reviewer for their thoughtful and constructive feedback. We are pleased that the reviewer recognizes the value of our open letter in emphasizing the urgent need for gender-sensitive reception policies for women migrants in Europe. We welcome the opportunity to clarify and improve our manuscript in response to the comments. Below, we address each point raised: **1. Incorporation of Empirical Data** We appreciate the reviewer’s recommendation to strengthen the article with more quantifiable data. Our original intention was to offer a qualitative and policy-focused perspective rooted in field observations and testimonies gathered through COST Action Women on the Move (CA19112). However, we agree that statistical references would enhance our arguments and have now added relevant data on GBV prevalence, health outcomes, and integration indicators where available in the revised version. We remain mindful that access to disaggregated and standardized data across centers remains limited, which is one of the key systemic weaknesses we highlight. **2. Detailed Methodology** We take note of the comment regarding the need for more methodological clarity. While the piece was conceived as a policy brief rather than a traditional academic article, we can clarify the following:

- Criteria for selection of the three reception centers : the reception centers do not select the migrants, yet the Spanish and Israel one focus on women, which is a gender-based selection criteria
- Description of the qualitative approach used : participant observation;
- The data analysis followed our thematic coding of field notes following our observations.

These should help readers better assess the validity and reliability of our conclusions. **3. Feasibility of Recommendations** We recognize the importance of providing more actionable steps for implementing the recommendations. We believe that the concrete examples of good practices drawn from the three case studies (e.g., Ödos in Spain, Usivak in Bosnia & Herzegovina, and Kutchinate in Israel), are relevant. Our intent is to encourage the adoption of best practices within realistic institutional constraints, while still pressing for structural change. **4. Critique of Policy Enforcement** We acknowledge the concern that too many acronyms and references to policy instruments may limit accessibility, and that enforcement gaps were not sufficiently analyzed. We believe the section on international and EU frameworks containt reduced jargon, and reflect the disconnection between policy formulation and practice. These critical insights are essential to push the debate beyond legal frameworks toward accountability. **Additional Points:**

- **Rationale for the Open Letter**: Our rationale is to emphasize the urgency of addressing gender-based vulnerabilities in reception systems and outline the limitations in existing practices that prompted the writing of this letter.
- **Differing Views and Factual Support**: While the brief presents a strong normative stance, we believe that we reflect differing institutional models and outcomes, supported by citations.
- **Accessibility**: We are pleased that the reviewer found the language clear and accessible. We continue to aim for inclusivity in tone and terminology to engage both academic and policy audiences.
- **Recommendations and Next Steps**: We organised recommendations to emphasize actionable items and align them with broader EU policy cycles, so that other researchers and stakeholders can engage with them.

We are grateful for the reviewer’s comments, which have enabled us to sharpen the analytical clarity, practical relevance, and transparency of our brief. We hope the revised version will better meet the expectations of both academic and policy audiences. With thanks and appreciation,

**Marie Ruiz & Stellamarina Donato**
